# Modulation of Cortical Inhibitory Circuits after Cathodal Transcranial Direct Current Stimulation over the Primary Motor Cortex

**DOI:** 10.3389/fnhum.2016.00030

**Published:** 2016-02-04

**Authors:** Ryoki Sasaki, Shota Miyaguchi, Shinichi Kotan, Sho Kojima, Hikari Kirimoto, Hideaki Onishi

**Affiliations:** Institute for Human Movement and Medical Sciences, Niigata University of Health and WelfareNiigata, Japan

**Keywords:** transcranial direct current stimulation, motor evoked potential, short-interval intracortical inhibition, short-latency afferent inhibition, primary motor cortex, primary somatosensory cortex

## Abstract

Here, we aimed to evaluate whether cathodal transcranial direct current stimulation (tDCS) over the primary motor cortex (M1) and primary somatosensory cortex (S1) can modulate cortical inhibitory circuits. Sixteen healthy subjects participated in this study. Cathodal tDCS was positioned over the left M1 (M1 cathodal) or left S1 (S1 cathodal) with an intensity of 1 mA for 10 min. Sham tDCS was applied for 10 min over the left M1 (sham). Motor evoked potentials (MEPs) elicited by transcranial magnetic stimulation (TMS) were recorded from the right abductor pollicis brevis (APB) muscle before the intervention (pre) and 10 and 30 min after the intervention (post 1 and post 2, respectively). Cortical inhibitory circuits were evaluated using short-interval intracortical inhibition (SICI) and short-latency afferent inhibition (SAI). M1 cathodal decreased single-pulse MEP amplitudes at post 1 and decreased SAI at post 1 and post 2; however, SICI did not exhibit any change. S1 cathodal and sham did not show any changes in MEP amplitudes at any of the three time points. These results demonstrated that cathodal tDCS over the M1 not only decreases the M1 excitability but also affects the cortical inhibitory circuits related to SAI.

## Introduction

Transcranial direct current stimulation (tDCS) is a noninvasive technique that allows the modulation of cortical excitability in humans (Priori, 2003). tDCS is capable of evoking excitability changes in the primary motor cortex (M1), and alterations in the induced excitability depend on the polarity, intensity, and duration of the applied stimulation (Nitsche and Paulus, 2000, 2001; Nitsche et al., 2003b). The M1 excitability is decreased by cathodal tDCS, whereas it is increased by anodal tDCS (Nitsche and Paulus, 2000). The mechanism of alterations in the tDCS-induced excitability remains poorly understood, but pharmacological studies have suggested that the effect of tDCS is attributed to immediate changes due to shifts in the membrane potential (Bindman et al., 1964) and after effects are induced by *N-methyl-D-aspartate* (NMDA) receptor activity modification (Liebetanz et al., 2002; Nitsche et al., 2003a). In currently used tDCS protocols, tDCS has been shown to modify cortical excitability lasting for approximately 1 h after the end of stimulation (Nitsche and Paulus, 2001; Nitsche et al., 2003b; Monte-Silva et al., 2010, 2013), raising the possibility that the physiological impact of tDCS involves synaptic plasticity, such as long-term depression- and long-term potentiation-like mechanisms (Liebetanz et al., 2002; Nitsche et al., 2003a).

Transcranial magnetic stimulation (TMS) is a safe, noninvasive, painless way to stimulate the human motor cortex, which can examine M1 excitability using motor evoked potentials (MEPs; Chen, 2000). The MEP amplitude elicited via TMS reflects M1 excitability. The MEP amplitude can be suppressed by the electrical stimulation of the median nerve when the interstimulus interval (ISI) between the median nerve stimulation and TMS is slightly longer than the latency of the N20 component of somatosensory evoked potentials (Tokimura et al., 2000). Such inhibition of the MEP is known as short-latency afferent inhibition (SAI), which is believed to involve cholinergic (Di Lazzaro et al., 2000b) and GABAergic systems (Di Lazzaro et al., 2005) at the level of the cortex (Tokimura et al., 2000). These reports raise the possibility that SAI is produced via the primary somatosensory cortex (S1) because the afferent input from the peripheral nerve arrives at S1. On the other hand, short-interval intracortical inhibition (SICI) is measured in a paired-pulse TMS paradigm, which is used at an ISI between the subthreshold conditioning pulse and suprathreshold test pulse of 1–5 ms (Kujirai et al., 1993). This inhibitory circuit is believed to occur at the cortical level (Kujirai et al., 1993; Di Lazzaro et al., 1998; Hanajima et al., 1998) through the activation of an intracortical inhibitory GABAergic system (Ziemann et al., 1996; Di Lazzaro et al., 2000a; Ilic et al., 2002). Several recent studies have shown the abnormal excitability of cortical inhibitory circuits in patients with Alzheimer’s disease (Di Lazzaro et al., 2002, 2004), Parkinson’s disease (Yarnall et al., 2013), and dystonia (Ridding et al., 1995). These studies demonstrated that these patients have reduced SICI or SAI, indicating the excitability of cortical inhibitory circuits.

tDCS can modulate the excitability of cortical inhibitory circuits involved in SICI and SAI. For example, SICI decreased following anodal tDCS over the M1 (Nitsche et al., 2005; Batsikadze et al., 2013; Kidgell et al., 2013) and SICI increased following cathodal tDCS over the M1 (Nitsche et al., 2005; Batsikadze et al., 2013). In addition, SAI increased following anodal tDCS over the M1 (Scelzo et al., 2011) and SAI decreased following cathodal tDCS over the S1 (Kojima et al., 2015). These results suggest that tDCS not only affect the M1 excitability but also affects the cortical inhibitory circuits. However, because there are few studies on cathodal tDCS than anodal tDCS, no conclusive evidence on the effect of cathodal tDCS on SICI and SAI has yet been reported.

The reduction of cortical excitability by cathodal tDCS may be useful for the treatment of neurological diseases associated with pathological cortical excitability enhancements (Nitsche et al., 2003b). Therefore, further research on the effects of cathodal tDCS is necessary. The aim of this study was to understand the effects of cathodal tDCS by investigating whether cathodal tDCS over the M1 and S1 can affect cortical inhibitory circuits. We hypothesized that cathodal tDCS over the M1 decreased the cortical inhibitory circuits related to SAI because anodal tDCS over the M1 increased SAI.

## Materials and Methods

### Subjects

Sixteen healthy subjects (12 males and 4 females; mean ± standard deviation, 21.6 ± 1.0 years; age range, 21–24 years) participated in this study. Fifteen subjects were right handed and one was left handed. None of the subjects was taking medications or had a history of physical, neurological, or psychiatric disorders. All subjects provided a written informed consent. This study was conducted in accordance with the Declaration of Helsinki, approved by the ethics committee of the Niigata University of Health and Welfare, and performed at the Institute for Human Movement and Medical Sciences (to which we belong). Furthermore, we explained the potential side effects of tDCS and TMS to all the subjects. The potential side effects during and after tDCS include pain, tingling, itching, and burning under the electrodes (Poreisz et al., 2007). In addition, the potential side effects of TMS include headache and local and neck pain because of the stimulation (Rossi et al., 2009). We decided to cancel an experiment immediately if the subject was not in a suitable condition.

### MEP Recording

MEPs were recorded from the abductor pollicis brevis (APB) muscle of the right hand using Ag–AgCl electrodes in a belly-tendon montage. Electromyography (EMG) signals were amplified (×100) by an amplifier (A-DL-720-140, 4 Assist, Tokyo, Japan), digitized (sampling rate, 4 kHz) using an A/D converter (Power Lab 8/30, AD Instruments, Colorado Springs, CO, USA), and filtered using a high-pass filter (20 Hz). Data were recorded on a computer and stored for analysis (LabChart7, AD Instruments). Moreover, an analysis software (Scope, AD Instruments) was used to measure the resting motor threshold (RMT).

Magnetic stimuli were delivered through a figure-of-eight coil (diameter, 9.5 cm) connected to two Magstim 200 stimulators via a Bistimu module (Magstim, Dyfed, UK). The coil was placed tangentially to the scalp, with the handle pointing posterolaterally at an approximate angle of 45° from the midline. The coil was positioned over the hand area of the left M1. An optimal coil position was selected so as to elicit the largest MEP in the right APB. Furthermore, the position and orientation of the coil was monitored throughout in individual magnetic resonance imaging (MRI) using Visor2 TMS Neuronavigation (eemagine Medical Imaging Solutions GmbH, Berlin, Germany). The hot spot of the APB muscle was recorded and the coil was manually held in place to maintain position. T1-weighted MRI was obtained using a 1.5-T system before the experiment (Signa HD, GE Healthcare, Milwaukee, WI, USA).

The RMT was determined as the minimum stimulus intensity that elicited MEPs (>50 μV in at least 5 of 10 trials) at rest. For obtaining single-pulse MEPs (single), the TMS intensity was adjusted to elicit 1 mV peak-to-peak amplitude (SI_1 mV_) at baseline. The RMT and SI_1 mV_ were expressed as a percentage of the maximum stimulator output (% MSO).

### SICI

SICI was studied using a paired TMS paradigm with a subthreshold conditioning stimulus followed by a suprathreshold test stimulus (Kujirai et al., 1993). The intensity of the conditioning stimulus was set at 80% of the RMT. SICI was evaluated at an ISI of 2 ms.

### SAI

Conditioning electrical pulses (0.2 ms square wave constant current pulses) were delivered through bar electrodes to the right median nerve at the wrist with the cathode positioned proximally (SEN-8203, Nihon Kohden, Tokyo, Japan). The stimulus intensity was adjusted to produce a slight thumb twitch in the thenar muscle of the right hand (Chen et al., 1999; Fischer and Orth, 2011). The ISI between the median nerve stimulation and TMS pulse was determined based on the latency of the N20m component of the somatosensory evoked magnetic fields (SEF) for each participant. The ISI was derived from the latency of the N20m component of the SEF and then an additional 2 ms was added (Tokimura et al., 2000). For SEF measurements, we used a 306-channel whole-head magnetoencephalography (MEG) system (Vectorview; Elekta, Helsinki, Finland). To elicit SEFs, the right median nerve was electrically stimulated with an intensity of the motor threshold (Neuropack Σ; Nihon Kohden, Tokyo, Japan).

### tDCS

**Figure 1:**
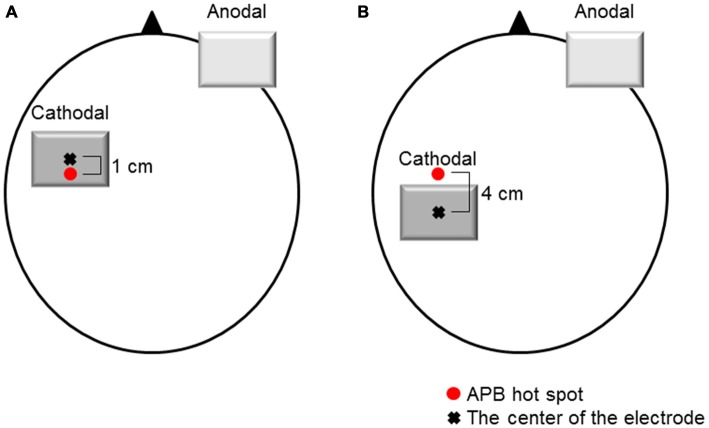
**Electrode positions. (A)** M1 cathodal. The cathodal electrode was positioned over the left M1. The left M1 was defined as the hot spot for the right abductor pollicis brevis (APB) muscle. The center of the cathodal electrode was placed 1.0 cm anterior to the hot spot of the APB muscle. **(B)** S1 cathodal. The cathodal electrode was positioned over the left S1. The left S1 was defined as 3.0 cm posterior to the hot spot for the right APB muscle. The center of the cathodal electrode was placed 4.0 cm posterior to the hot spot of the APB muscle. The anodal electrode was placed above the contralateral orbit.

tDCS was delivered by a direct current stimulator (Eidith, NeuroConn GmbH, Germany) using a saline-soaked pair of sponge electrodes (5 × 7 cm, 35 cm^2^). Cathodal tDCS was applied for 10 min (fade-in/fade-out time, 5 s) at 1.0 mA (current density, 0.029 mA/cm^2^). When cathodal tDCS was applied over the M1 (M1 cathodal), the center of the cathodal electrode was placed 1.0 cm anterior to the hot spot of the APB muscle to avoid covering the S1 area with the tDCS electrode (Figure 1A). When cathodal tDCS was applied over the left S1 (S1 cathodal), the left S1 was defined as being 3.0 cm posterior to the hot spot of the APB muscle (Koch et al., 2006). The center of the cathodal electrode was placed 4.0 cm posterior to the motor hot spot of the APB muscle to avoid covering the M1 area with the tDCS electrode (Figure 1B). When procedures were identical to the M1 cathodal, the current was switched off after 30 s of stimulation as sham condition (Gandiga et al., 2006). In all cases, the anodal electrode was placed above the contralateral orbit.

### Experimental Procedures

**Figure 2:**
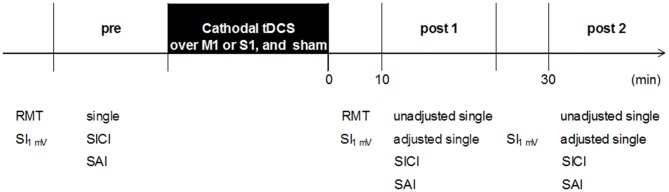
**Experimental procedure.** Sixteen subjects participated in the three experiments (M1 cathodal, S1 cathodal, and sham). The three experiments were performed in a repeated measurement design using a randomized order. The RMT was recorded before and after the intervention. Transcranial magnetic stimulation (TMS) parameters (single, SICI, and SAI) were performed in a randomized order: baseline (pre), 10 min after the intervention (post 1), and 30 min after the intervention (post 2). RMT, resting motor threshold; SI_1 mV_, TMS intensity to produce 1 mV; single, single-pulse MEPs; unadjusted single, single-pulse MEPs by unadjusted TMS intensity; adjusted single, single-pulse MEPs by adjusted TMS intensity; SICI, short-interval intracortical inhibition; SAI, short-latency afferent inhibition.

The experimental procedures are shown in Figure 2. Subjects were seated in a comfortable reclining chair with a mounted headrest during experiments. All subjects received blinded stimulation of tDCS and the three tDCS conditions were randomly applied to the subjects. On the other hand, the researcher was not blinded to the tDCS stimulation because it was necessary to confirm the tDCS settings and correct positioning by two researchers. The session interval was ≥3 days, which was sufficient to confirm no influence of cathodal tDCS. Cathodal tDCS was applied over the left M1 or left S1, and sham was applied over the left M1. After determination of the hot spot of the APB muscle, the RMT was obtained. The hot spot of the APB muscle was identified by TMS, and the stimulation intensity was adjusted to elicit single-pulse MEPs of 1 mV. Subsequently, the three parameters (single, SICI, and SAI) were recorded 12 times each (0.2 Hz) before the intervention (pre). These parameters were measured in a randomized order by controlling the pulse control system (Pulse Timer II, Medical Try System, Tokyo, Japan). After the intervention, the RMT was measured immediately and the other parameters (single, SICI, and SAI) were measured in approximately 10 min (post 1) and 30 min (post 2) after the intervention. For single-pulse MEP measurements, the same stimulus intensity was used before and after the intervention (unadjusted single). In addition, the single-pulse MEP intensity was readjusted at each time point to elicit test MEPs of 1 mV (adjusted single), if needed. After adjustment for single-pulse MEPs, other parameters (unadjusted single, adjusted single, SICI, and SAI) were measured in a randomized order.

### Data Analysis

The mean MEP amplitudes, with the maximum and minimum MEP amplitudes excluded, were calculated from the peak-to-peak amplitudes of 10 trials in each of the TMS parameters (single, SICI, and SAI).

Statistical analysis was performed using PASW statistics software version 18 (SPSS; IBM, Armonk, NY, USA). Two-way repeated measures analysis of variance (ANOVA) was used to analyze MEP amplitudes and SI_1 mV_. The factors for the ANOVA were three interventions [factor: INTERVENTION (M1 cathodal, S1 cathodal, and sham)] and three time points [factor: TIME (pre, post 1, and post 2)]. Bonferroni’s methods were used for *post hoc* comparisons. The level of significance was set at *P* < 0.05.

## Results

### TMS Intensity to Produce 1 mV (SI_1 mV_)

Two-way repeated-measures ANOVA revealed significant interaction effect of INTERVENTION × TIME [*F*_(4,60)_ = 8.228, *P* = 0.000], whereas no significant changes were found for the main effects of INTERVENTION [*F*_(2,30)_ = 0.497, *P* = 0.613] and TIME [*F*_(2,30)_ = 2.344, *P* = 0.113]. *Post hoc* analyses showed that after the M1 cathodal, SI_1 mV_ significantly increased between at post 1 (*P* = 0.000) and post 2 (*P* = 0.014) compared with that at pre (pre, 58.8 ± 7.7%; post 1, 60.6 ± 7.9%; post 2, 60.0 ± 7.8%). After the S1 cathodal (pre, 59.6 ± 7.1%; post 1, 58.9 ± 7.8%; post 2, 59.7 ± 7.5%) and sham (pre, 59.2 ± 7.7%; post 1, 59.2 ± 7.7%; post 2, 59.2 ± 7.7%), SI_1 mV_ was not significantly different among the three time points.

### Single-Pulse MEPs by Unadjusted TMS Intensity (Unadjusted Single)

**Figure 3:**
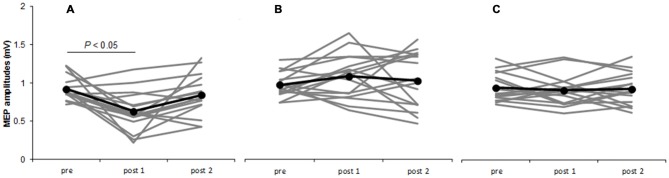
**Time courses of MEP amplitudes.** M1 cathodal **(A)** S1 cathodal **(B)** sham **(C)** on single-pulse MEP amplitudes by unadjusted TMS intensity. Gray bars indicate individual MEP amplitudes. Black bars indicate mean MEP amplitudes. At the M1 cathodal, compared with baseline values, MEP amplitudes significantly decreased at post 1 (*P* < 0.05) but not at post 2. At the S1 cathodal and sham, no significant changes were observed following any of the interventions.

Two-way repeated-measures ANOVA revealed significant main effects of INTERVENTION [*F*_(2,30)_ = 10.277, *P* = 0.000] and interaction effect of INTERVENTION × TIME [*F*_(4,60)_ = 4.225, *P* = 0.004], whereas no significant changes were found for the main effect of TIME [*F*_(2,30)_ = 2.192, *P* = 0.129]. *Post hoc* analyses showed that after the M1 cathodal, MEP amplitudes significantly decreased at post 1 compared with that at pre (*P* = 0.015), but MEP amplitudes at post 2 did not exhibit any change (*P* = 0.832). After the S1 cathodal and sham, MEP amplitudes were not significantly different among the three time points (Figure 3, Table 1).

**Table 1:** **Mean values of single, SICI, and SAI before and after the three interventions**.

	M1 cathodal			S1 cathodal			Sham		
	Pre	Post 1	Post 2	Pre	Post 1	Post 2	Pre	Post 1	Post 2
Unadjusted single	0.92 ± 0.04	0.63 ± 0.06^*^	0.84 ± 0.07	0.98 ± 0.04	1.09 ± 0.07	1.03 ± 0.09	0.95 ± 0.05	0.91 ± 0.05	0.93 ± 0.05
Adjusted single	0.92 ± 0.04	0.93 ± 0.05	0.91 ± 0.04	0.98 ± 0.04	0.96 ± 0.04	1.03 ± 0.05			
SICI	0.34 ± 0.06	0.41 ± 0.09	0.40 ± 0.05	0.34 ± 0.06	0.39 ± 0.06	0.43 ± 0.08	0.41 ± 0.08	0.38 ± 0.09	0.41 ± 0.09
SAI	0.51 ± 0.08	0.84 ± 0.14^**^	0.77 ± 0.10^*^	0.64 ± 0.08	0.81 ± 0.13	0.80 ± 0.15	0.59 ± 0.08	0.45 ± 0.09	0.55 ± 0.09

Unadjusted single, single-pulse MEPs by unadjusted TMS intensity; adjusted single, single-pulse MEPs by adjusted TMS intensity; SICI, short-interval intracortical inhibition; SAI, short-latency afferent inhibition. Unadjusted single, adjusted single, SICI, SAI; mean ± standard error ^*^P < 0.05, ^**^P < 0.01.

### Single-Pulse MEPs by Adjusted TMS Intensity (Adjusted Single)

Two-way repeated-measures ANOVA showed that no significant main effects of INTERVENTION [*F*_(2,30)_ = 1.300, *P* = 0.287] and TIME [*F*_(2,30)_ = 0.426, *P* = 0.528], and no significant changes were found for the interaction effect of INTERVENTION × TIME [*F*_(4,60)_ = 0.803, *P* = 0.165] (Table 1).

### SICI

Two-way repeated-measures ANOVA showed that no significant main effect of INTERVENTION [*F*_(2,30)_ = 0.037, *P* = 0.964] and TIME [*F*_(1.390, 20.850)_ = 0.753, *P* = 0.438], and no significant changes were found for the interaction effect of INTERVENTION × TIME [*F*_(4,60)_ = 0.463, *P* = 0.763] (Table 1).

### SAI

Two-way repeated-measures ANOVA revealed significant main effect of INTERVENTION [*F*_(2,30)_ = 3.796, *P* = 0.034] and interaction effect of INTERVENTION × TIME [*F*_(4,60)_ = 4.101, *P* = 0.005], whereas no significant changes were found for the main effect of TIME [*F*_(2,30)_ = 3.235, *P* = 0.053]. *Post hoc* analyses revealed that after the M1 cathodal, MEP amplitudes significantly increased between at post 1 (*P* = 0.003) and post 2 (*P* = 0.018) compared with that at pre. After the S1 cathodal and sham, MEP amplitudes were not significantly different among the three time points (Table 1).

## Discussion

Our results indicated that the cathodal tDCS at an intensity of 1 mA over M1 decreases M1 excitability and excitability of cortical inhibitory circuits related to SAI, whereas no change was observed in the SICI. In addition the cathodal tDCS over S1 did not affect the M1 excitability and excitability of cortical inhibitory circuits related to SICI and SAI.

Cathodal tDCS over the M1 has been shown to decrease the M1 excitability for more than an hour (Nitsche et al., 2003b; Monte-Silva et al., 2010; Di Lazzaro et al., 2012). It is suggested that cortical excitability shifts induced by tDCS depend on the modulation of the resting membrane potential (Bindman et al., 1964) and the after effects are NMDA receptor-dependent (Liebetanz et al., 2002; Nitsche et al., 2003a). Therefore, it is suggested that the effects of cathodal tDCS result from the reduction of the resting membrane potential or reduction in NMDA receptor activity.

Cathodal tDCS over M1 does not affect SICI. Di Lazzaro et al. (2012) found that cathodal tDCS at an intensity of 1 mA does not affect SICI, whereas Nitsche et al. (2005) and Batsikadze et al. (2013) found that cathodal tDCS at an intensity of 1 mA increased SICI. These inconsistencies are believed to be related to differences in the conditioning stimulus intensity used in SICI. Di Lazzaro et al. (2012) measured SICI using the conditioning stimulus intensity of 95% of the active motor threshold (AMT), whereas Nitsche et al. (2005) and Batsikadze et al. (2013) measured SICI using the conditioning stimulus intensity of 70% of the AMT. Several studies have shown that the magnitude of SICI suppression depends on the intensity of the conditioning stimulus (Ilic et al., 2002; Vucic et al., 2009), and the best suppression is observed with a conditioning stimulus intensity of 70–90% of the RMT (Kujirai et al., 1993). In this study, SICI was examined using the conditioning stimulus intensity of 80% of the RMT leading to maximum suppression. However, Nitsche et al. (2005) evaluated SICI with a weak conditioning stimulus intensity (70% of the AMT) compared with that used in this study. This weak conditioning stimulus intensity was selected because the inhibitory effect of the conditioning stimulus was too strong. Nitsche et al. (2005) used a weak conditioning stimulus intensity in which the inhibitory effects of SICI were slight. In contrast, in the present study and in the study by Di Lazzaro et al. (2012), high conditioning stimulus intensity was used to measure SICI. It is considered that the effects of cathodal tDCS did not change SICI because of the strong inhibitory effects of SICI in this study.

The muscarinic receptor antagonist scopolamine decreases SAI in healthy subjects (Di Lazzaro et al., 2000b). Moreover, SAI also decreases in patients with Alzheimer’s disease and increases when these patients are administered acetylcholinesterase inhibitors (Di Lazzaro et al., 2002, 2004). In addition, a GABA_A_ receptor agonist reduced SAI (Di Lazzaro et al., 2005, 2007). These results indicate that cholinergic and GABAergic systems are involved in the inhibitory circuits of SAI. In the study of effects of tDCS, anodal tDCS over the M1 significantly increased SAI (Scelzo et al., 2011); therefore, anodal tDCS can not only increase the M1 excitability but also function to enhance cortical inhibitory circuits in SAI. Additionally, a pharmacological study indicated that a cholinesterase inhibitor blocked the induction of excitability changes associated with tDCS (Kuo et al., 2007). This finding raises the possibility that cholinergic systems involve cortical excitability changes induced by tDCS. On the basis of the results of the present study, cathodal tDCS contributes to the reduction in SAI by affecting cortical inhibitory circuits, including cholinergic systems and GABAergic systems.

Cathodal tDCS applied to the S1 decreased the N20 source component of somatosensory evoked potentials (Dieckhofer et al., 2006), which implies that it affects the afferent input to the S1 elicited by the stimulation of the median nerve. The median nerve afferent input arrives at the S1. Therefore, the cortical inhibitory circuits of SAI may be generated via the S1. We believed that the changes in the S1 excitability would affect SAI if SAI is caused via a cortico-cortical pathway from the S1 to M1. However, SAI did not significantly change after cathodal tDCS. Therefore, we could not determine the pathway through which SAI is produced. The first excitatory cortical response from the area 3b (N20m), indicating the S1 excitability, was unaffected by the administration of the muscarinic antagonist scopolamine (Huttunen et al., 2001); however, SAI decreased (Di Lazzaro et al., 2000b). Consequently, there is no relationship between the S1 excitability and SAI changes. These findings support the results of this study that cathodal tDCS applied over the S1 did not affect SAI. In contrast, Kojima et al. (2015) have shown that SAI decreased when cathodal tDCS was applied over the S1. This result differs from that of our study. There are three differences between this study and that of Kojima et al. (2015). First, in this study, cathodal tDCS at intensity (current density) was different compared with that in the study by Kojima et al. (2015). Kojima et al. (2015) used the cathodal tDCS at an intensity of 1 mA same as that used in this study. However, the electrode surface area is smaller than that used in the present study. Consequently, cathodal tDCS at that intensity was high compared with that in this study. Second, a method of peripheral nerve stimulation for SAI was different between the studies. Kojima et al. (2015) stimulated the index finger at an intensity three times the perceptual sensory threshold without muscle contraction, whereas we stimulated the median nerve at an intensity of motor threshold, which caused a slight thumb twitch. Third, the ISI between the electrical stimulation and TMS is different between studies. Kojima et al. (2015) set the ISI at 40 ms, whereas we adopted an ISI that added 2 ms to the peak of N20m. In the study of paired associative stimulation (PAS), Hamada et al. (2012) reported that two different mechanisms are involved in the effects using PAS at different ISIs (PAS_21.5 ms_ vs. PAS_25 ms_). Similarly, it is possible that SAI inhibits M1 in different pathways by the differences in ISI. These may influence one of these three or all differences. Cathodal tDCS over S1 does not affect SICI. The cortical inhibitory circuits of SICI result from the synaptic inhibition within the motor cortex (Di Lazzaro et al., 1998). In addition, previous studies indicated that PAS and theta-burst stimulation on S1 did not alter SICI (Krivanekova et al., 2011; Jacobs et al., 2014); therefore, cathodal tDCS over the S1 does not affect the cortical inhibitory circuits of SICI, similar to these previous studies.

There are several limitations associated with this study. The results of the present study differed from those of previous studies because cathodal tDCS over M1 and S1 did not affect SICI and SAI, respectively, in this study. We considered several factor; however, further studies are necessary. In future, we wish to conduct studies by setting plural factors (e.g., effect of conditioning stimulus intensity on SICI, tDCS intensity, effect of the ISI on SAI, and a method to stimulate the peripheral nerve for SAI). Next, this study involved small groups of subjects. Several studies showed that the effects of tDCS are highly variable when using anodal tDCS and cathodal tDCS at 2 mA for 10 min (Wiethoff et al., 2014) or anodal tDCS at 1 mA for 13 min (López-Alonso et al., 2015). However, our results clearly indicated that cathodal tDCS over M1 decreased M1 excitability; however, sham tCDS showed no effects.

In conclusion, the present study shows that cathodal tDCS over the M1 decreases the M1 excitability and excitability of cortical inhibitory circuits related to SAI but it does not affect the excitability of cortical inhibitory circuits related to SICI. In addition, cathodal tDCS over the S1 does not affect the M1 excitability or excitability of cortical inhibitory circuits related to SAI and SICI.

## Author Contributions

HO conceived the study and designed the experiment. RS and SM conducted the experiments. SK aand HK performed interpretation of data. RS and SK performed the statistical analysis. SM, SK and HK helped writing and revising the manuscript. HO and RS wrote the manuscript. All authors read and approved the final manuscript.

## Conflict of Interest Statement

The authors declare that the research was conducted in the absence of any commercial or financial relationships that could be construed as a potential conflict of interest.
